# Atropine or Cyclopentolate to Diagnose Premyopia in Preschool Children

**DOI:** 10.1001/jamaophthalmol.2025.3243

**Published:** 2025-09-25

**Authors:** Haotian Wu, Yanjiao Wang, Qiuying Lu, Kathryn A. Rose, Ian G. Morgan, Zihan Ni, Kaidi Xiang, Ziyi Qi, Bo Zhang, Jingjing Wang, Jun Chen, Xun Xu, Xiangui He

**Affiliations:** 1 Shanghai Eye Diseases Prevention and Treatment Center/Shanghai Eye Hospital, School of Medicine, Tongji University, Shanghai, China; 2 Department of Ophthalmology, Shanghai General Hospital, Shanghai Jiao Tong University School of Medicine, National Clinical Research Center for Eye Diseases, Shanghai, China; 3 Discipline of Orthoptics, Graduate School of Health, University of Technology Sydney, Ultimo, New South Wales, Australia; 4 Division of Biochemistry and Molecular Biology, Research School of Biology, Australian National University, Canberra, Australian Capital Territory, Australia; 5 Department of Ophthalmology, The Affiliated People’s Hospital of Ningbo University, The Eye Hospital of Wenzhou Medical University (Ningbo Branch), Ningbo, China

## Abstract

**Importance:**

Sufficient cycloplegia is essential for reliable refraction in preschool children. The choice of cycloplegic agent may affect refraction and diagnosis outcomes.

**Objective:**

To evaluate the objective refraction outcomes after cycloplegia in preschool children given either atropine or cyclopentolate.

**Design, Setting, and Participants:**

This was a post hoc analysis of the 2024 Preschool Children Refractive Development Pattern and Influencing Factors Study (PRDP-IFS), in which children were given atropine, and the 2013 to 2014 Elaborative Shanghai Childhood Ocular Refractive Development Study (E-SCORDS), in which children were given cyclopentolate. These were population-based studies. Eyes in each group were included via propensity score matching. Study data were analyzed from December 2024 to July 2025.

**Exposures:**

Cycloplegia induced by either 1% atropine (twice daily for 4 days with an additional dose on day 5) or 1% cyclopentolate (dual administration 5 minutes apart) eye drops.

**Main Outcomes and Measures:**

Difference between the noncycloplegic and cycloplegic spherical equivalent (DSE) and the prevalence of refractive states were compared in the atropine and cyclopentolate groups. Refractive states (moderate to high hyperopia, low hyperopia, premyopia, and myopia) were determined by cycloplegic spherical equivalent (SE) using an autorefractor.

**Results:**

A total of 1761 children and their 3048 eyes were included in this study. There were 773 children (1524 eyes) in the atropine group (mean [SD] age, 4.62 [0.92] years; 406 male [52.5%]) and 988 children (1524 eyes) in the cyclopentolate group (mean [SD] age, 4.62 [0.93] years; 530 male [53.6%]). There were a total of 1524 eyes in each group. The mean (SD) noncycloplegic SE was 0.30 (0.92) diopters (D) and 0.31 (0.76) D in the atropine and cyclopentolate groups, respectively (mean difference, −0.01 D; 95% CI, −0.07 to 0.05 D; *P* = .72). Mean (SD) DSE in the atropine group was 1.56 (0.72) D and in the cyclopentolate group was 0.97 (0.70) D. The mean difference in DSE between the 2 groups was 0.59 D (95% CI, 0.54-0.64 D; *P* < .001). The difference in the percentages of refractive states between the atropine and cycloplegic groups was as follows: moderate to high hyperopia (7.2% vs 2.7% = 4.5%; 95% CI, 2.9%-6.0%; *P* < .001), low hyperopia (82.8% vs 74.0% = 8.8%; 95% CI, 6.0%-11.8%; *P* < .001), premyopia (8.7% vs 21.6% = −12.9%; 95% CI, −15.4% to −10.4%; *P* < .001), and myopia (1.3% vs 1.8% = −0.5%; 95% CI, −1.3% to 0.4%; *P* = .30).

**Conclusions and Relevance:**

This study found that use of atropine for cycloplegia in preschool children was associated with less myopic refraction compared with cyclopentolate and may potentially avoid overestimation of premyopia prevalence; however, this investigation did not evaluate each cycloplegic agent in the same children.

## Key Points

**Question:**

How do cycloplegic refraction outcomes differ in preschool children given either atropine or cyclopentolate?

**Findings:**

In a post hoc analysis of 2 population-based studies, 1 from 2013 to 2014 (cyclopentolate group) and the other from 2024 (atropine group), among a total of 1761 children aged 3 to 7 years and 3048 eyes, after cycloplegia, the cyclopentolate group showed more myopic spherical equivalent, a higher prevalence of premyopia, and a lower prevalence of hyperopia compared with the atropine group.

**Meaning:**

This study found that the use of atropine for cycloplegia in preschool children was associated with less myopic refraction compared with cyclopentolate and potentially avoids overestimation of premyopia prevalence; however, this investigation did not evaluate each cycloplegic agent in the same children.

## Teaser

This comparative effectiveness study investigates cycloplegic refraction outcomes in preschool children given either atropine or cyclopentolate.

## Introduction

Comprehensive eye examinations including cycloplegic refraction are essential for diagnosing and managing common pediatric ocular conditions, especially in children who fail vision screening.^1^ Due to strong accommodation in children, cycloplegia is necessary to minimize accommodation-induced errors and obtain reliable refraction outcomes.^1,2,3^ Meanwhile, with myopia increasingly recognized as a major global public health concern, the usage of cycloplegic eye drops has become more widespread to facilitate the identification of children with myopia or premyopia and support early intervention.^1,4,5^

The preferred cycloplegic agents vary across countries and clinical settings.^1,2,6,7^ Although atropine is the strongest cycloplegic agent and was recommended for first-time refraction in children younger than 6 years in China, its use in routine practice is limited due to its prolonged effects, potential adverse effects, and administration inconvenience.^2^ Cyclopentolate, with its more rapid onset and shorter duration than atropine, is frequently used as an alternative in children.^8^ However, there is insufficient evidence regarding whether cyclopentolate achieves cycloplegia comparable with atropine in preschool-aged children, and more research is needed to quantify the differences in cycloplegic refraction outcomes with different agents.^9^

This study aimed to determine how cycloplegic refraction outcomes differed in preschool children aged 3 to 7 years in a post hoc analysis of 2 different population-based studies from 2013 to 2014 (cycloplegic refraction with cyclopentolate group) and 2024 (cycloplegic refraction with atropine group).

## Methods and Materials

### Study Participants

The atropine group (cycloplegic refraction with atropine) was obtained from the baseline data of the Preschool Children Refractive Development Pattern and Influencing Factors Study (PRDP-IFS), conducted in Shanghai, China, between March and June 2024, with the approval by the Ethics Committee of the Shanghai Eye Disease Prevention and Treatment Center. We included all children aged 3 to 7 years who completed cycloplegia and underwent objective refraction both before and after cycloplegia. Ethnicity information was obtained from school registration records according to the study protocol and included Han, Hui, Manchu, Tujia, Yao, and Zhuang ethnicities.

The cyclopentolate group (cycloplegic refraction with cyclopentolate) was first obtained from the baseline data of the Elaborative Shanghai Childhood Ocular Refractive Development Study (E-SCORDS), a population-based study conducted between September 2013 and October 2014.^10,11^ We included all children aged 3 to 7 years who completed cycloplegia and had refraction data both before and after cycloplegia. This dataset was then matched to the atropine group using propensity score matching (PSM), yielding a final sample set of eyes.

Detailed inclusion and exclusion criteria and participant number for the PRDP-IFS and E-SCORDS studies are provided in the eMethods and eTable 4 in Supplement 1. Both the PRDP-IFS and E-SCORDS studies adhered to the Declaration of Helsinki. Written informed consent was obtained from the parents or guardians of all participants. All examinations were provided free of charge. Although no monetary stipend was offered to participants, small gifts such as school supplies were provided to encourage participation. This study followed the International Society for Pharmacoeconomics and Outcomes Research (ISPOR) reporting guidelines.^12^

### Ophthalmic Examinations

In the PRDP-IFS study, noncycloplegic and cycloplegic refraction were performed using an autorefractor (KR-8900 [Topcon]). Axial length (AL) was measured with the IOL Master 700, version 5.02 (Carl Zeiss Meditec). Intraocular pressure was assessed using noncontact tonometry (NT-4000 [Nidek Inc]), and a slitlamp biomicroscope (Carl Zeiss) was used to examine the anterior segment. Examination methods in the E-SCORDS study have been described previously.^10,11^

### Cycloplegia Procedure

In the atropine group, cycloplegia was induced using 1% atropine sulfate eye gel (Dishan [Xingqi Co Ltd]). Atropine was instilled twice daily (9:30 am and 2:30 pm) for 4 consecutive days, with a final dose given on the morning of the fifth day.^2^ Cycloplegia was considered adequate if pupil diameter reached greater than 6 mm and there was an absence of direct pupil response 1 hour later. Children were inquired daily during atropine use regarding symptoms such as blurred near vision or photophobia, along with assessments of pupil size and whether facial flushing occurred. One month after cycloplegia, parents were contacted by telephone to confirm whether these symptoms had disappeared. In the cyclopentolate group, cycloplegia was induced with 1 eye drop of 0.5% proparacaine hydrochloride in each eye, followed by 2 eye drops of 1.0% cyclopentolate (Cyclogyl [Alcon]) at a 5-minute interval. Pupil size and direct pupil response were assessed 30 minutes later; if criteria were unmet, an additional eye drop was given.

### Statistical Analysis

The difference between noncycloplegic and cycloplegic spherical equivalent (DSE) was defined as cycloplegic spherical equivalent (SE) minus noncycloplegic SE (NSE). Hyperopia was classified by cycloplegic SE as follows: low (0.75 diopters [D]< SE <3.00 D), moderate (3.00 D≤ SE <5.00 D), and high (SE ≥5.00 D).^13^ Premyopia was defined as −0.50 D less than SE less than or equal to 0.75 D, and myopia was defined as SE less than or equal to −0.50 D.^14^

The cyclopentolate group was derived using PSM to reduce potential confounding with the atropine group. Specifically, a logistic regression model was applied to estimate propensity scores based on key baseline characteristics, including age, grade, sex, noncycloplegic spherical and cylindrical power, and AL. Participants in the atropine group were matched 1:1 with those in the E-SCORDS study using nearest-neighbor matching without replacement, applying a caliper width of 0.2 SDs of the logit of the propensity score to minimize residual bias.^15^

Quantitative data were presented as mean (SD), whereas categorical data were expressed as percentages. Categorical, ordinal, and continuous variables were compared using the χ^2^ test, the Cochran-Armitage test, or *t* test. Statistical analyses were performed using R software, version 4.4.3 (R Project for Statistical Computing). Statistical significance was set at a 2-tailed *P* value <.05, and data analysis was performed from December 2024 to July 2025.

## Results

### Propensity Score Matching and Characteristics of Participants

A total of 1761 children and their 3048 eyes were included in this study. All the atropine group samples were successfully matched in the PSM procedure, with no cases discarded (PSM model output were provided in eTable 1 in Supplement 1). There were 773 children (1524 eyes) in the atropine group (mean [SD] age, 4.62 [0.92] years; 367 female [47.5%]; 406 male [52.5%]) and 988 children (1524 eyes) in the cyclopentolate group (mean [SD] age, 4.62 [0.93] years; 458 female [46.4%]; 530 male [53.6%]), with a mean difference of 0 year (95% CI, −0.07 to 0.07 years; *P* = .98). Among the 1761 participants, 1750 (99.4%) were identified as Han Chinese. In the atropine group, 767 participants (99.2%) were Han, 1 (0.1%) was Hui, 2 (0.3%) were Manchu, 1 (0.13%) was Tujia, 1 (0.1%) was Yao, and 1 (0.1%) was Zhuang. In the cyclopentolate group, 883 participants (99.5%) were Han Chinese, 1 (0.1%) was Manchu, 3 (0.3%) were Tujia, and 1 (0.1%) was Zhuang.

From the E-SCORDS study (cyclopentolate group), we initially included 5678 eyes, which was then matched to the atropine group yielding a final sample of 1524 eyes in each group. The mean (SD) NSE was 0.30 (0.92) D and 0.31 (0.76) D in the atropine and cyclopentolate groups, respectively, with a mean difference of −0.01 D (95% CI, −0.07 to 0.05 D; *P* = .72). Other demographic and ocular characteristics of study participants were presented in Table 1. No differences were observed between the right and left eyes in either group.

**Table 1.** Participants’ Characteristics Stratified by the Atropine and Cyclopentolate Groups

Characteristic	Atropine group	Cyclopentolate group	Mean difference^a^	95% CI^b^	*P* value^b^
No. of eyes	1524	1524	NA	NA	NA
Right eye, No. (%)	763 (50.1)	782 (51.3)	NA	NA	.51
Left eye, No. (%)	761 (49.9)	742 (48.7)	NA	NA	
Sex, No. (%)					
Female	367 (47.5)	458 (46.4)	NA	NA	.10
Male	406 (52.2)	530 (53.6)	NA	NA	
Age, mean (SD), y	4.62 (0.92)	4.62 (0.93)	0	(−0.07 to 0.07)	.98
Grade, No. (%)					
Junior class	509 (33.4)	502 (32.9)	NA	NA	.14
Middle class	529 (34.7)	537 (35.2)			
Senior class	486 (31.9)	485 (31.8)			
IOP, mean (SD), mm Hg	13.56 (2.58)	13.66 (2.65)	−0.10	(−0.28 to 0.09)	.35
AL, mean (SD), mm	22.36 (0.73)	22.36 (0.74)	−0.01	(−0.06 to 0.05)	.84
CR, mean (SD), mm	7.85 (0.25)	7.85 (0.27)	0	(−0.02 to 0.02)	.87
UCVA, mean (SD), logMAR [Snellen]	0.20 (0.13) [20/32]	0.20 (0.11) [20/32]	0	(−0.01 to 0.01)	.59
BCVA, mean (SD), logMAR [Snellen]	0.18 (0.11) [20/30]	0.16 (0.08) [approximately 20/29]	0.02	(0.01 to 0.02)	<.001
Noncycloplegic refraction, mean (SD), D					
Spherical power	0.55 (0.96)	0.56 (0.80)	−0.01	(−0.07 to 0.06)	.84
Cylindrical power	−0.50 (0.52)	−0.50 (0.60)	−0.01	(−0.05 to 0.03)	.67
SE	0.30 (0.92)	0.31 (0.76)	−0.01	(−0.07 to 0.05)	.72
Cycloplegic refraction, mean (SD), D					
Spherical power	2.12 (1.10)	1.54 (0.86)	0.58	(0.51 to 0.65)	<.001
Cylinder power	−0.52 (0.53)	−0.52 (0.61)	0.00	(−0.04 to 0.04)	.95
SE	1.86 (1.06)	1.28 (0.83)	0.58	(0.51 to 0.65)	<.001
DSE	1.56 (0.72)	0.97 (0.70)	0.59	(0.54 to 0.64)	<.001

Abbreviations: AL, axial length; BCVA, best-corrected visual acuity; CR, corneal curvature radius ratio; D, diopter; DSE, difference of noncycloplegic and cycloplegic spherical equivalent; IOP, intraocular pressure measured by a noncontact tonometry; NA, not applicable; SE, spherical equivalent, calculated as the sum of the spherical power and half of the cylindrical power; UCVA, uncorrected visual acuity.

^a^ The difference in indicators between the 2 groups, calculated by subtracting the value of the cyclopentolate group from that of the atropine group.

^b^ Between-group comparisons using an unpaired *t* test to calculate 95% CIs and *P* values.

### Differences in Objective Refraction Outcomes After Cycloplegia

**Figure 1.**
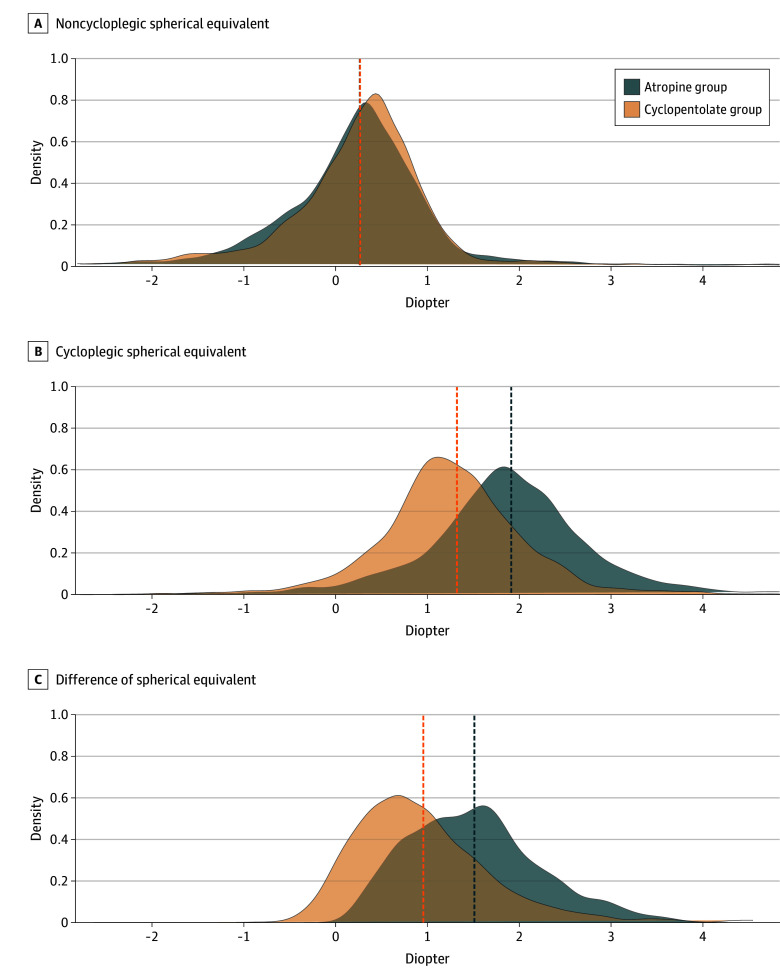
Density Plots of Noncycloplegic Spherical Equivalent (SE), Cycloplegic SE, and Difference of Noncycloplegic and Cycloplegic SE (DSE) in the Atropine and Cyclopentolate Groups Dashed lines indicate group means. The noncycloplegic SE was 0.30 (0.92) diopter (D) in the atropine group and 0.31 (0.76) D in the cyclopentolate group, with a mean difference of −0.01 D (95% CI, −0.07 to 0.05 D; *P* = .72). Cycloplegic SE was 1.86 (1.06) D in the atropine group and 1.28 (0.83) D in the cyclopentolate group (95% CI, 0.51-0.65 D; *P* < .001). DSE was 1.56 (0.72) D in the atropine group and 0.97 (0.70) D in the cyclopentolate group, with a mean difference of 0.59 D (95% CI, 0.54-0.64 D; *P* < .001).

After cycloplegia, the atropine group showed higher cycloplegic SE than the cyclopentolate group (95% CI, 0.51-0.65 D; *P* < .001). Mean (SD) DSE was 1.56 (0.72) D in the atropine group, and 0.97 (0.70) D in the cyclopentolate group, with a mean difference of 0.59 D (95% CI, 0.54-0.64 D; *P* < .001). Figure 1 presented the density distributions of NSE, cycloplegic SE, and DSE in both groups. Scatterplots of noncycloplegic and cycloplegic SE and DSE in the atropine and cyclopentolate groups were shown as eFigures 1 and 2 in Supplement 1.

**Figure 2.**
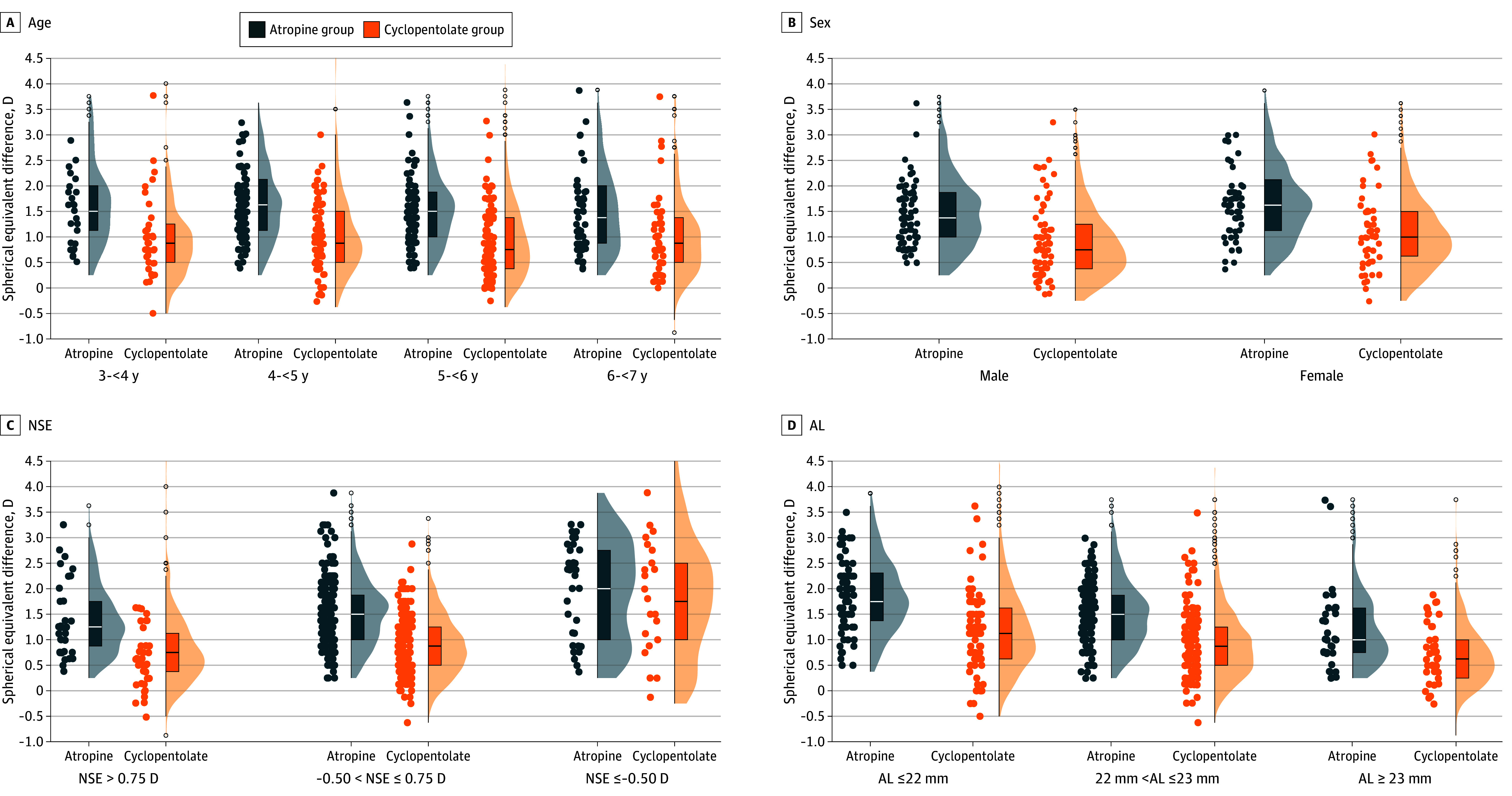
Subgroup Analysis of the Difference of Noncycloplegic and Cycloplegic Spherical Equivalent (DSE) Between the Atropine and Cyclopentolate Groups The DSE was calculated as cycloplegic SE minus noncycloplegic SE. The left half of the figures presented scatterplots, and the right half combined box plots and density curves. AL indicates axial length; D, diopter; NSE, noncycloplegic SE.

Participants were further stratified by various demographic and ocular characteristics for subgroup analysis. Full results are presented in eTable 2 in Supplement 1. Differences in DSE between the atropine and cyclopentolate groups were observed in all subgroups, whereas only participants with NSE less than or equal to −0.50 D exhibited a mean DSE difference of less than or equal to 0.25 D between the atropine and cyclopentolate groups. DSE difference between the atropine and cyclopentolate groups in the subgroups stratified by age, sex, NSE, and AL were demonstrated as Figure 2.

### Differences in the Percentages of Refractive States

Participants in both groups were classified into refractive states based on their cycloplegic SE with either atropine or cyclopentolate, including moderate to high hyperopia, low hyperopia, premyopia, and myopia. Moderate to high hyperopia was observed in 7.2% of the atropine group and 2.7% of the cyclopentolate group, with a mean difference of 4.5% (95% CI, 2.9%-6.0%; *P* < .001). Low hyperopia was observed in 82.8% of the atropine group and 74.0% of the cyclopentolate group, with a mean difference of 8.8% (95% CI, 6.0%-11.8%; *P* < .001). The prevalence of premyopia was 8.7% in the atropine group and 21.6% in the cyclopentolate group, with a mean difference of −12.9% (95% CI, −15.4% to −10.4%; *P* < .001). The prevalence of myopia was 1.3% in the atropine group and 1.8% in the cyclopentolate group, with a mean difference of −0.5% (95% CI, −1.3% to 0.4%; *P* = .30).

**Figure 3.**
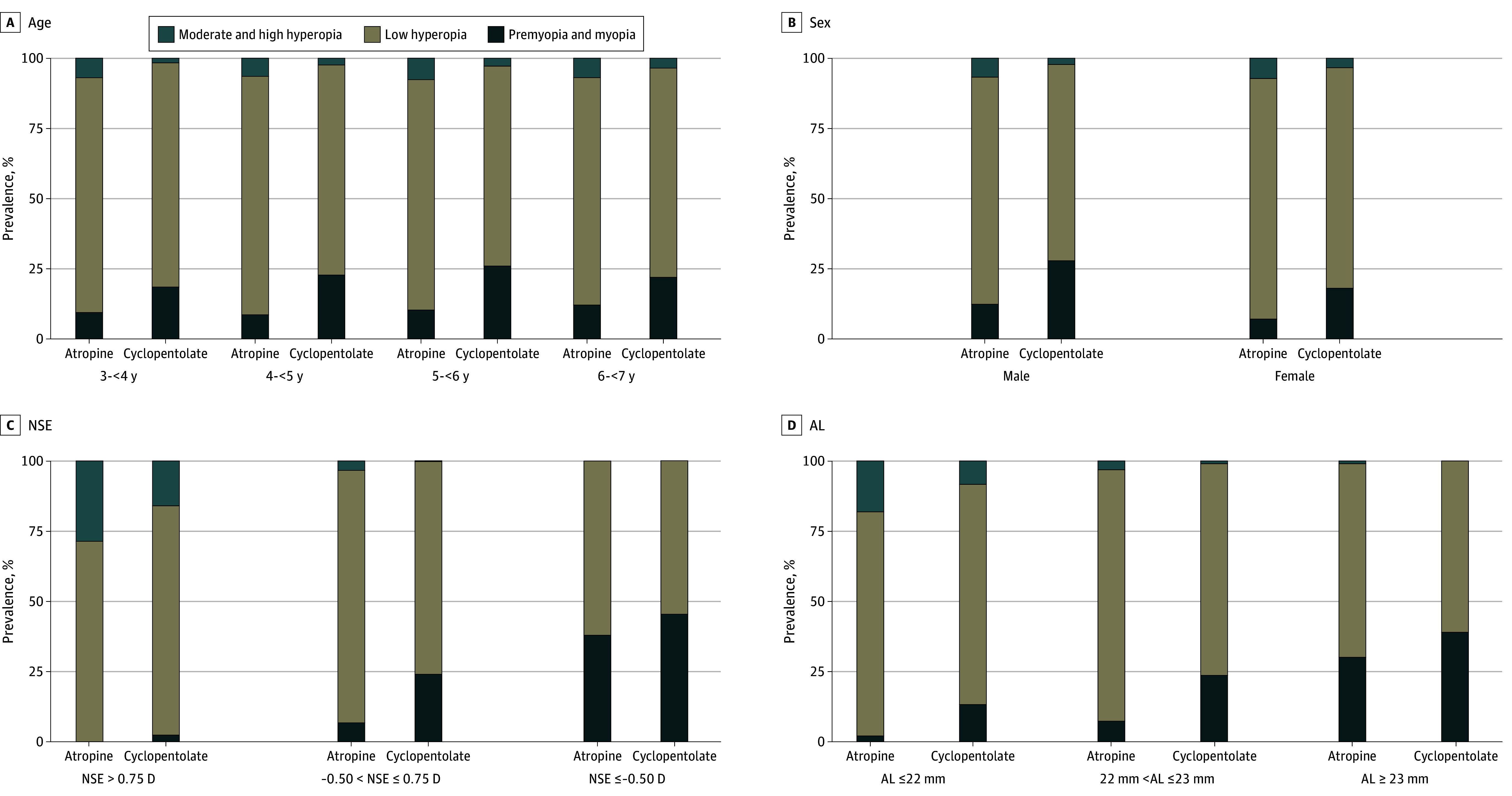
Subgroup Analysis of Difference in Prevalence of Refractive States Between the Atropine and Cyclopentolate Groups Refractive states were determined based on cycloplegic spherical equivalent (SE) obtained using either atropine or cyclopentolate. AL indicates axial length; D, diopter; NSE, noncycloplegic SE.

Subgroup analysis was conducted based on participants’ demographic and ocular characteristics. The number and percentage of different refractive states in different subgroups were provided in Table 2, and the results of subgroup analysis stratified by age, sex, NSE, and AL were shown as Figure 3. Differences in the percentage of refractive states were observed between the atropine and cyclopentolate groups across most subgroups, except for participants with NSE less than or equal to −0.50 D (*z* = −1.63; *P* = .10) and AL greater than 23 mm (*z* = −1.84; *P* = .06).

**Table 2.** Difference in Prevalence of Refractive States Between the Atropine and Cyclopentolate Groups and Subgroup Analysis

Variable	No. (%)								
	Atropine group				Cyclopentolate group				*P* value^**a**^
	Moderate and high hyperopia	Low hyperopia	Premyopia	Myopia	Moderate and high hyperopia	Low hyperopia	Premyopia	Myopia	
Total	109 (7.2)	1262 (82.8)	133 (8.7)	20 (1.3)	41 (2.7)	1127 (74.0)	329 (21.6)	27 (1.8)	<.001
Age, y									
3-<4	11 (6.9)	133 (83.6)	13 (8.2)	2 (1.3)	3 (1.7)	143 (79.4)	32 (17.8)	2 (1.1)	.003
4-<5	39 (6.8)	484 (84.5)	44 (7.7)	6 (1.0)	13 (2.5)	391 (74.6)	111 (21.2)	9 (1.7)	<.001
5-<6	37 (7.7)	395 (82.0)	48 (10.0)	2 (0.4)	15 (2.8)	378 (71.2)	127 (23.9)	11 (2.1)	<.001
6-<7	22 (7.1)	250 (80.6)	28 (9.0)	10 (3.2)	10 (3.5)	215 (74.4)	59 (20.4)	5 (1.7)	.005
Sex									
Female	53 (7.3)	617 (85.3)	47 (6.5)	6 (0.8)	24 (3.4)	560 (78.8)	124 (17.4)	3 (0.4)	<.001
Male	56 (7.0)	645 (80.5)	86 (10.7)	14 (1.7)	17 (2.1)	567 (69.7)	205 (25.2)	24 (3.0)	<.001
NSE, D									
>0.75	76 (28.6)	190 (71.4)	0	0	38 (15.9)	195 (81.6)	6 (2.5)	0	<.001
>−0.50 to ≤0.75	33 (3.1)	943 (89.9)	73 (7.0)	0	3 (0.3)	832 (75.6)	264 (24.0)	2 (0.2)	<.001
≤−0.50	0	129 (61.7)	60 (28.7)	20 (9.6)	0	100 (54.3)	59 (32.1)	25 (13.6)	.10
AL, mm									
≤22	88 (17.9)	393 (80.0)	10 (2.0)	0	36 (7.7)	369 (79.0)	61 (13.1)	1 (0.2)	<.001
>22 to ≤23	19 (2.6)	670 (90.1)	51 (6.9)	4 (0.5)	5 (0.6%)	590 (75.5)	176 (22.5)	10 (1.3)	<.001
>23	2 (0.7)	199 (68.9)	72 (24.9)	16 (5.5)	0	168 (60.9)	92 (33.3)	16 (5.8)	.06
AL/CR ratio									
≤2.8	93 (28.9)	228 (70.8)	1 (0.3)	0	31 (10.2)	239 (78.9)	31 (10.2)	2 (0.7)	<.001
>2.8 to ≤2.9	16 (1.8)	807 (92.7)	47 (5.4)	1 (0.1)	9 (1.0)	729 (81.1)	156 (17.4)	5 (0.6)	<.001
>2.9	0	227 (68.6)	85 (25.7)	19 (5.7)	1 (0.3)	159 (49.4)	142 (44.1)	20 (6.2)	<.001

Abbreviations: AL/CR, axial length/corneal curvature radius (calculated as axial length divided by the corneal curvature radius); D, diopter; NSE, noncycloplegic spherical equivalent.

^a^Comparison of refractive states prevalence between atropine and cyclopentolate groups was performed using the χ^2^ test. Between-group comparisons were performed using Cochran-Armitage test to determine *P* values.

## Discussion

Based on 2 population-based studies, we examined differences in cycloplegic refraction outcomes among preschool children aged 3 to 7 years who received either atropine or cyclopentolate. In summary, after cycloplegia, the cyclopentolate group showed more myopic SE, a higher prevalence of premyopia, and a lower prevalence of low hyperopia and moderate to high hyperopia compared with the atropine group. Differences in DSE and the percentages of refractive states between groups remained consistent across most subgroups. Although children in the atropine and cyclopentolate groups were similar in age, noncycloplegic refraction and AL after PSM, those given atropine exhibited more myopic cycloplegic refraction and had a lower prevalence of premyopia compared with those given cyclopentolate. These findings suggest that using atropine for cycloplegia in preschool children yields less myopic refraction than cyclopentolate, potentially avoiding overestimation of premyopia prevalence.

The preference of cycloplegic agents varies across regions, influenced by clinician preferences, drug availability, and population-specific evidence. Atropine provides the strongest cycloplegic efficacy; however, it also has the slowest onset, the longest duration of effect, and is more likely to induce systemic adverse effects.^16,17^ Due to the trade-off between its strong cycloplegic efficacy and practical inconvenience, atropine is recommended in China for first-time refraction in younger children, whereas in some other guidelines, it is considered unnecessary.^1,2,6,7^ In most cases, cyclopentolate is the preferred alternative for use in children due to its faster onset and shorter duration of action, allowing to complete cycloplegic refraction in the same day.^8,18^ Studies comparing atropine and cyclopentolate have yielded inconsistent results due to variations in age and refraction of the study population, as well as differences in study design, with limited generalizability due to small sample sizes. A parallel-designed interventional study^19^ of 67 children (aged 4-17 years) found only a 0.18-D difference between the 2 agents, whereas a self-controlled prospective study^20^ of 80 Chinese children (aged 4-9 years) reported a mean (SD) cycloplegic SE of 2.22 (3.52) D with atropine and 1.74 (3.46) D with cyclopentolate, with a mean (SD) difference of 0.48 (0.46) D.

In our study, the mean DSE difference between the atropine and cyclopentolate groups was 0.59 D (95% CI, 0.54-0.64 D), with the DSE in the cyclopentolate group accounting for only 62.2% of that in the atropine group. Despite similar NSE between the 2 groups, differences in DSE led to divergent cycloplegic SE outcomes. The mean (SD) cycloplegic SE was 1.86 (1.06) D in the atropine group and 1.28 (0.83) D in the cyclopentolate group. Notably, a Chinese expert consensus based on multiple epidemiological studies using cyclopentolate for cycloplegia, reported an average hyperopic reserve of 1.38 D in 6- to 7-year-old children.^21^ This value more closely resembles that of the preschool children in our cyclopentolate group but differs largely from the corresponding value observed in the atropine group.

The prevalence of refractive states also differed between the atropine and cyclopentolate groups, as categorized based on cycloplegic SE. Notably, the prevalence of premyopia was 8.7% in the atropine group, and 21.6% in the cyclopentolate group, more than twice that observed in the atropine group. The prevalence of hyperopia also declined accordingly in the cyclopentolate group, with a between-group difference of 4.5% for moderate to high hyperopia (7.2% vs 2.7%) and 8.8% for low hyperopia (82.8% vs 74.0%). It is worth noting that no clear difference in myopia prevalence was found between the atropine (1.3%) and cyclopentolate (1.8%) groups (*P* = .30). This may be attributed to the small number of myopic children in both groups or to the diminishing discrepancy between cycloplegic agents with increasing degrees of myopia.

A subgroup analysis was conducted based on known demographic and ocular influencing factors associated with DSE.^22,23,24,25^ The DSE difference between atropine and cyclopentolate decreased with age, from 0.71 D at 3 to 4 years to 0.50 D at 6 to 7 years. However, even among children aged 6 to 7 years, which is the upper limit of our study population, discrepancy remained beyond the commonly accepted threshold. Our findings also suggest that only in the subgroup of preschool children with NSE less than or equal to −0.50 D was the intergroup DSE difference less than 0.25 D. Differences in premyopia prevalence were observed across most subgroups, except among those defined by NSE less than or equal to −0.50 D (*P* = .45), as shown in eTable 3 in Supplement 1.

Children with premyopia have a high likelihood of progressing to myopia within 1 to 2 years, and the fastest rate of change in refractive error and AL occurred during the year before myopia onset rather than in any year after onset.^14,26,27,28,29^ Meanwhile, growing evidence suggests that early intervention in children with premyopia can effectively reduce the incidence of myopia and slow myopic shift.^30,31,32,33^ Thus, timely identification and management of premyopia are becoming increasingly important. The observed intergroup differences in refractive state prevalence suggest that the choice of cycloplegic agent may lead to inconsistent classification—particularly in identifying premyopia. Such discrepancies may result in unnecessary or delayed interventions, potentially hindering efforts to prevent myopia.

In our study, we used PSM to establish comparable groups for analysis rather than adopting a within-subject comparison approach, given the practical challenges of recruiting participants willing to undergo 2 separate cycloplegic procedures with a short time interval—particularly 1 involving atropine. Although the atropine and cyclopentolate groups showed similar mean characteristics after PSM, their underlying distributions were not fully equivalent. It is also important to note that the cyclopentolate group after PSM represents a reconstructed dataset rather than a representative subset of the original population. Its demographic and refractive characteristics may differ from those of the source dataset, as the matching process used the atropine group as the reference. Additionally, the nearly decade-long gap between the 2 source datasets further complicates data interpretation. These considerations underscore limitations of the PSM approach, which may introduce bias to intergroup comparisons.

In our study, the primary outcome was defined as the difference between noncycloplegic and cycloplegic refraction, through which we identified disparities between the atropine and cyclopentolate groups. However, direct measurements of accommodation—such as residual accommodative tone—were not performed, limiting our ability to further investigate the underlying causes of the observed intergroup DSE differences. This was because the present study was not a primary outcome or a prespecified secondary analysis of either the E-SCORDS or PRDP-IFS study and accommodation measurement was not included in the protocol of the 2 studies. Although the observed differences in DSE may mainly be attributed to the varying abilities of atropine and cyclopentolate to paralyze the accommodative system, atropine may also influence cycloplegic refraction outcomes through additional mechanisms, such as changes in choroidal thickness and lens power.^9^ More research is needed to further elucidate this issue.

In the PRDP-IFS study, all children experienced varying degrees of photophobia and near vision blur after receiving atropine. This inconvenience led to only approximately half of the sampled children obtaining parental consent for cycloplegia, and just 44.9% completed the 5-day regimen (shown as eTable 4 in Supplement 1). Most recovered within 1 to 3 weeks, although 2 children reported symptoms lasting over 1 month. Flush and fever were the most common adverse events. Most symptoms resolved within 2 to 3 hours after hydration and increased urination. No severe adverse events or allergic reactions were observed. Although exact data on photophobia and near vision blur were unavailable in the E-SCORDS study, no cycloplegia-related adverse events were reported in previous publications.^10,11^ As neither the 2 studies were primarily designed to evaluate the safety and efficacy of cycloplegic agents, the reported adverse effect data may be of lower evidentiary strength.^17,34^ Neither study included objective assessments of photophobia or near blur (eg, photopic pupil size or near visual acuity), relying instead on subjective observations, self-reports, and follow-up phone interviews.

Given the inconvenience associated with atropine administration and its differing cycloplegic outcomes compared with cyclopentolate in preschool children, it is important to better understand which children are most likely to benefit from atropine-induced cycloplegia. It remains uncertain whether the observed differences in cycloplegic refraction outcomes would influence clinical decision-making. This uncertainty stems from the limited understanding of which agent yields a more accurate diagnosis of premyopia and better identifies children who may benefit from early myopia intervention. Future longitudinal data may help address this question by comparing refractive development and treatment responses among children diagnosed with premyopia using different cycloplegic agents.

### Limitations

Additional limitations of our study should be noted. First, our findings were based solely on Chinese preschool children, which may limit generalizability to other populations, as iris pigmentation and ethnicity have been shown to influence the efficacy and duration of cycloplegic agents.^16,35^ Second, our study did not include a comparison between atropine and tropicamide due to the lack of suitable data, although tropicamide is also commonly used in clinical settings and has been reported to exhibit comparable effectiveness to cyclopentolate in several studies.^36,37,38,39^ Lastly, children with amblyopia, strabismus, or other ocular conditions were excluded, preventing the evaluation of cycloplegic responses in these subgroups.

## Conclusions

In this comparative effectiveness study, use of atropine in preschool children for cycloplegia was associated with in less myopic refraction compared with cyclopentolate and may potentially avoid the overestimation of premyopia prevalence, although this investigation did not evaluate each cycloplegic agent in the same children.

## Notes

Supplement 1. **eMethods.** Inclusion and Exclusion Criteria for the PRDP-IFS and E-SCORDS Studies **eTable 1.** Summary of the Propensity Score Matching (PSM) Results **eTable 2.** Cycloplegic Refraction Differences Between the Atropine and Cyclopentolate Groups and Subgroup Analysis Results **eTable 3.** Difference of Premyopia Prevalence Between the Atropine and Cyclopentolate Groups and Subgroup Analysis Results **eTable 4.** Number and Percentage of Children Who Consented to and Completed Cycloplegia **eFigure 1.** Scatterplot of DSE and Cycloplegic SE Using Either Atropine or Cyclopentolate **eFigure 2.** Scatterplot of Noncycloplegic and Cycloplegic SE Using Either Atropine or Cyclopentolate

Supplement 2.
Data Sharing Statement.
