# Supplementary material for: Atropine or Cyclopentolate to Diagnose Premyopia in Preschool Children
Source: JAMA Ophthalmol. 2025 Sep 25;143(11):904–13. doi: 10.1001/jamaophthalmol.2025.3243 (PMC12464850; doi:10.1001/jamaophthalmol.2025.3243)
Supplement: Supplement 1. — eMethods. Inclusion and Exclusion Criteria for the PRDP-IFS and E-SCORDS Studies eTable 1. Summary of the Propensity Score Matching (PSM) Results eTable 2. Cycloplegic Refraction Differences Between the Atropine and Cyclopentolate Groups and Subgroup Analysis Results eTable 3. Difference of Premyopia Prevalence Between the Atropine and Cyclopentolate Groups and Subgroup Analysis Results eTable 4. Number and Percentage of Children Who Consented to and Completed Cycloplegia eFigure 1. Scatterplot of DSE and Cycloplegic SE Using Either Atropine or Cyclopentolate eFigure 2. Scatterplot of Noncycloplegic and Cycloplegic SE Using Either Atropine or Cyclopentolate [file jamaophthalmol-e253243-s001.pdf]

Supplementary Online Content

Wu H, Wang Y, Lu Q, et al. Atropine or cyclopentolate to diagnose premyopia in preschool children. *JAMA Ophthalmol*. Published online September 25, 2025. doi:10.1001/jamaophthalmol.2025.3243

eMethods. Inclusion and Exclusion Criteria for the PRDP-IFS and E-SCORDS Studies

eTable 1. Summary of the Propensity Score Matching (PSM) Results

eTable 2. Cycloplegic Refraction Differences Between the Atropine and Cyclopentolate Groups and Subgroup Analysis Results

eTable 3. Difference of Premyopia Prevalence Between the Atropine and Cyclopentolate Groups and Subgroup Analysis Results

eTable 4. Number and Percentage of Children Who Consented to and Completed Cycloplegia

eFigure 1. Scatterplot of DSE and Cycloplegic SE Using Either Atropine or Cyclopentolate

eFigure 2. Scatterplot of Noncycloplegic and Cycloplegic SE Using Either Atropine or Cyclopentolate

This supplementary material has been provided by the authors to give readers additional information about their work.

© 2025 Wu H et al. *JAMA Ophthalmology*.

eMethods: The inclusion criteria for the PRDP-IFS study were: (1) children aged 3-7 years enrolled in kindergarten; (2) intraocular pressure ≤ 21 mmHg; and (3) peripheral anterior chamber depth > 1/4 corneal thickness, assessed using the Van Herick method. Exclusion criteria included: (1) strabismus, amblyopia or other ocular diseases; (2) systemic diseases potentially affecting the eyes; and (3) known contraindications to atropine, including glaucoma, epilepsy or other central nervous system disorders, tachyarrhythmias or congenital heart disease, and being allergic to atropine. For the E-SCORDS study, inclusion criteria included enrollment in kindergarten and absence of any history of foreign ethnicity, cataract, glaucoma, or retinopathy. Children attending schools specifically designated for migrant populations were excluded. Additionally, children with a personal or family history of epilepsy or other neurological disorders were excluded from undergoing cycloplegia.

© 2025 Wu H et al. *JAMA Ophthalmology*.

eTable 1. Summary of the propensity score matching (PSM) results

	Means Treated*	Means Control†	SMD	Var. Ratio	eCDF Mean	eCDF Max	Std. Pair Dist.
**Comparison between atropine and cyclopentolate groups before PSM**							
Distance	0.39	0.30	0.65	1.12	0.174	0.310	-
Age	4.62	4.38	0.26	1.00	0.061	0.097	-
Grade							
Junior class	0.33	0.31	0.06	-	0.028	0.028	-
Middle class	0.35	0.34	0.02	-	0.010	0.010	-
Senior class	0.32	0.36	-0.08	-	0.038	0.038	-
Gender							
Male	0.47	0.48	-0.01	-	0.004	0.004	-
Female	0.53	0.52	0.01	-	0.004	0.004	-
Non-cycloplegic refraction							
Spherical power	0.55	0.58	-0.02	1.14	0.011	0.060	-
Cylindrical power	-0.50	-0.48	-0.05	0.85	0.018	0.178	-
AL	22.36	22.27	0.12	0.94	0.021	0.057	-
**Comparison between atropine and cyclopentolate groups after PSM**							
Distance	0.39	0.39	0.02	1.08	0.005	0.037	0.034
Age	4.62	4.62	0.00	0.98	0.008	0.015	0.835
Grade							
Junior class	0.33	0.33	0.02	-	0.012	0.012	0.876
Middle class	0.35	0.35	-0.02	-	0.022	0.022	0.944
Senior class	0.32	0.32	0.02	-	0.010	0.010	0.753
Gender							
Female	0.47	0.47	-0.02	-	0.010	0.010	0.996
Male	0.53	0.53	0.02	-	0.010	0.010	0.996
Non-cycloplegic refraction							
Spherical power	0.55	0.55	-0.03	0.94	0.009	0.052	0.907
Cylindrical power	-0.50	-0.50	-0.02	0.72	0.020	0.178	0.913
AL	22.36	22.36	0.01	0.93	0.006	0.031	1.084

"SMD": standardized mean difference; "Var. Ratio": variance ratio; "eCDF": empirical cumulative distribution function; "Std. Pair Dist.": Standardized Pairwise Distance.

*: Mean value of the variants among atropine group.

†: Mean value of the variants among E-SCORDS data before PSM and cyclopentolate group

© 2025 Wu H et al. *JAMA Ophthalmology*.

eTable 2. Cycloplegic refraction differences between the atropine and cyclopentolate groups and subgroup analysis

	Atropine group		Cyclopentolate group		Mean difference, D*	95% CI†	*P* values†
	N	DSE, D	N	DSE, D			
Total	1524	1.56 (0.72)	1524	0.97 (0.70)	0.59	(0.54 to 0.64)	<.001
Age							
3-4 yrs.	159	1.63 (0.78)	180	0.92 (0.64)	0.71	(0.56 to 0.87)	<.001
4-5 yrs.	573	1.62 (0.69)	524	1.02 (0.70)	0.60	(0.52 to 0.69)	<.001
5-6 yrs.	482	1.52 (0.67)	531	0.93 (0.71)	0.59	(0.50 to 0.67)	<.001
6-7 yrs.	310	1.49 (0.78)	289	0.99 (0.71)	0.50	(0.38 to 0.62)	<.001
Gender							
Boys	801	1.49 (0.71)	813	0.89 (0.69)	0.60	(0.54 to 0.67)	<.001
Girls	723	1.64 (0.71)	711	1.06 (0.70)	0.58	(0.50 to 0.65)	<.001
Non-cycloplegic SE, D							
nSE > 0.75	266	1.37 (0.64)	239	0.80 (0.63)	0.57	(0.46 to 0.68)	<.001
-0.50<nSE<=0.75	1049	1.54 (0.65)	1101	0.89 (0.58)	0.65	(0.59 to 0.70)	<.001
nSE <= -0.50	209	1.92 (0.98)	184	1.67 (0.99)	0.24	(0.05 to 0.44)	.02
Axial length							
AL <= 22	491	1.86 (0.70)	467	1.18 (0.75)	0.67	(0.58 to 0.77)	<.001
22<AL<=23	744	1.51 (0.65)	781	0.93 (0.67)	0.58	(0.51 to 0.64)	<.001
AL > 23	289	1.19 (0.70)	276	0.71 (0.57)	0.48	(0.38 to 0.59)	<.001
Axial length/corneal radius of curvature ratio							
AL/CR <= 2.8	322	2.02 (0.69)	303	1.20 (0.70)	0.82	(0.71 to 0.93)	<.001
2.8< AL/CR<= 2.9	871	1.56 (0.65)	899	1.02 (0.69)	0.54	(0.48 to 0.60)	<.001
AL/CR > 2.9	331	1.12 (0.62)	322	0.62 (0.57)	0.50	(0.41 to 0.59)	<.001
Refractive status							
Moderate & high hyperopia	109	2.32 (0.76)	41	1.75 (0.88)	0.57	(0.24 to 0.9)	<.001
Low hyperopia	1262	1.60 (0.65)	1127	1.11 (0.65)	0.49	(0.44 to 0.54)	<.001
Pre-myopia	133	0.74 (0.37)	329	0.47 (0.55)	0.28	(0.20 to 0.36)	<.001
Myopia	20	0.58 (0.24)	27	0.20 (0.38)	0.38	(0.19 to 0.56)	<.001

"nSE": Non-cycloplegic spherical equivalent. "AL/CR": The axial length/corneal curvature radius ratio (AL/CR) was calculated as axial length divided by the corneal curvature radius.

*: Mean difference in DSE between the atropine and cyclopentolate group, calculated by subtracting the value of the cyclopentolate group from that of the atropine group.

†: Between-group comparisons of DSE using an unpaired t-test to calculate 95% confidence intervals and *P* values.

© 2025 Wu H et al. *JAMA Ophthalmology*.

eTable 3. Difference in pre-myopia prevalence between the atropine and cyclopentolate groups and subgroup analysis

	Atropine group		Cyclopentolate group		Mean difference*	95% CI†	*P* values†
	N	Pre-myopia	N	Pre-myopia			
Total	1524	133 (8.7%)	1524	329 (21.6%)	-12.9%	(-15.4% to -10.4)	<.001
Age							
3-4 yrs.	159	13 (8.2%)	180	32 (17.8%)	-9.6%	(-16.6% to -2.6%)	.01
4-5 yrs.	573	44 (7.7%)	524	111 (21.2%)	-13.5%	(-17.6% to -9.4%)	<.001
5-6 yrs.	482	48 (10.0%)	531	127 (23.9%)	-14.0%	(-18.5% to -9.5%)	<.001
6-7 yrs.	310	28 (9.0%)	289	59 (20.4%)	-11.4%	(-17% to -5.7%)	<.001
Gender							
Boys	801	86 (10.7%)	813	205 (25.2%)	-14.5%	(-18.2% to -10.8%)	<.001
Girls	723	47 (6.5%)	711	124 (17.4%)	-10.9%	(-14.3% to -7.6%)	<.001
Non-cycloplegic SE, D							
nSE > 0.75	266	0 (0.0%)	239	6 (2.5%)	-2.5%	(-5.4% to -1.1%)	.01
-0.50<nSE<=0.75	1049	73 (7.0%)	1101	264 (24.0%)	-17%	(-20.0% to -14.1%)	<.001
nSE <= -0.50	209	60 (28.7%)	184	59 (32.1%)	-3.4%	(-12.5% to 5.8%)	.45
Axial length							
AL <= 22	491	10 (2.0%)	467	61 (13.1%)	-11%	(-14.3% to -7.7%)	<.001
22<AL<=23	744	51 (6.9%)	781	176 (22.5%)	-15.7%	(-19.1% to -12.2%)	<.001
AL > 23	289	72 (24.9%)	276	92 (33.3%)	-8.4%	(-15.9% to -1.0%)	<.001
Axial length/corneal radius of curvature ratio							
AL/CR <= 2.8	322	1 (0.3%)	303	31 (10.2%)	-9.9%	(-13.9% to -6.0%)	<.001
2.8< AL/CR<= 2.9	871	47 (5.4%)	899	156 (17.4%)	-12.0%	(-14.9% to -9.1%)	<.001
AL/CR > 2.9	331	85 (25.7%)	322	142 (44.1%)	-18.4%	(-25.6% to -11.2%)	<.001

"nSE": Non-cycloplegic spherical equivalent. "AL/CR": The axial length/corneal curvature radius ratio (AL/CR) was calculated as axial length divided by the corneal curvature radius.

*: Represents the mean intergroup difference in pre-myopia prevalence between the atropine and cyclopentolate groups

†: Between-group comparisons of pre-myopia prevalence were initially performed using chi-square tests to calculate 95% confidence intervals and *P* values. In cases with observed frequencies <5, the Newcombe-Wilson method was employed to compute 95% CIs, and Fisher's exact test was used to determine *P* values.

© 2025 Wu H et al. *JAMA Ophthalmology*.

eTable 4. Number and percentage of children who consented to and completed cycloplegia

Studies	Sampled children, n (%)	Consented to cycloplegia, n (%)	Completed cycloplegia, n (%)
PRDP-IFS	2478 (100.0%)	1242 (50.1%)	1113 (44.9%)
E-SCORDS*	4617 (100.0%)	2891 (62.6%)	2851 (61.8%)

*: Data for E-SCORDS were obtained from a previously published study (Zhang, 2018).

© 2025 Wu H et al. *JAMA Ophthalmology*.

eFigure 1. Scatterplot of difference of non-cycloplegic and cycloplegic SE (DSE) and cycloplegic SE using different agents

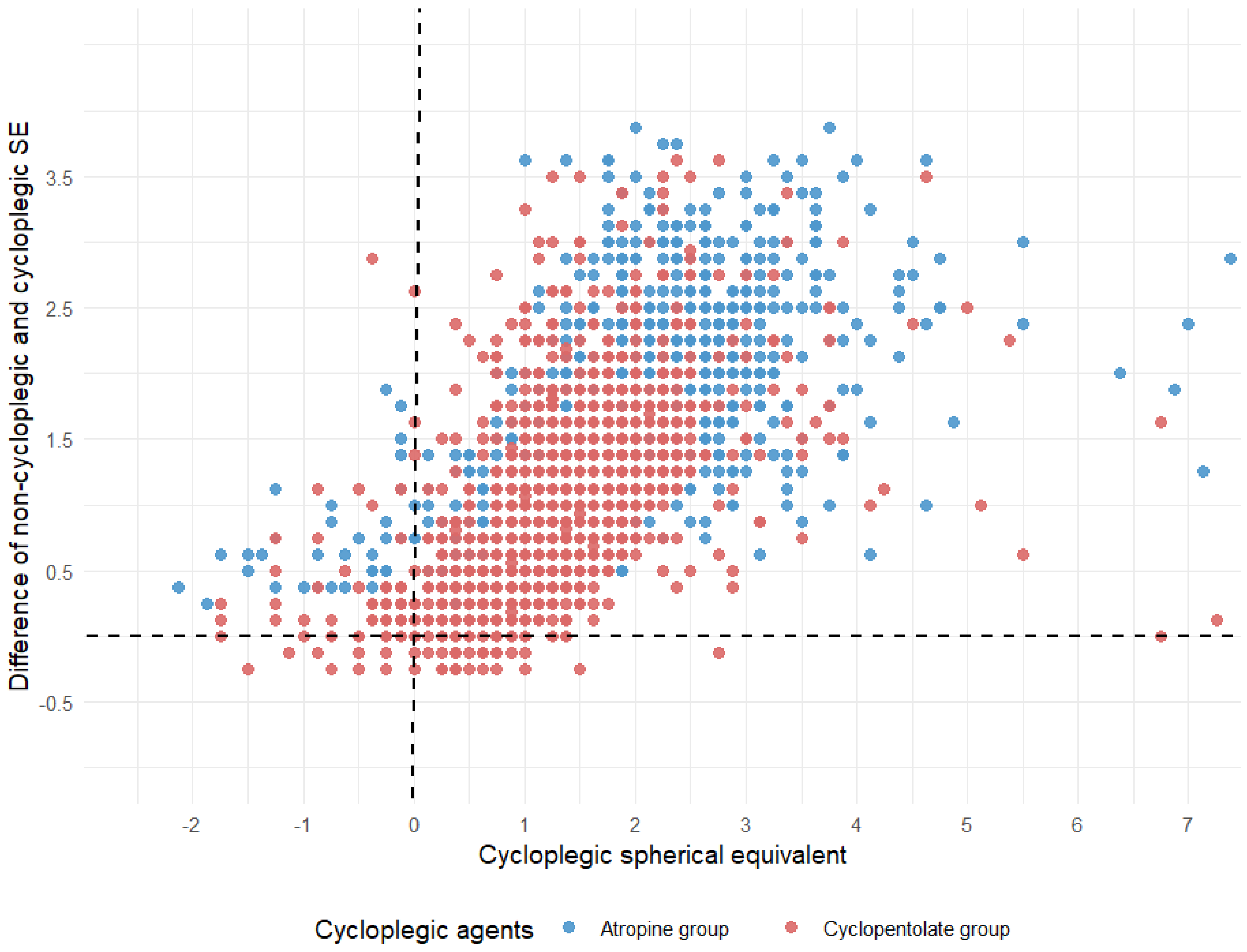

The dashed reference lines in the figure represent Y = 0 (horizontal axis) and X = 0 (vertical axis) respectively.

© 2025 Wu H et al. *JAMA Ophthalmology*.

eFigure 2. Scatterplot of non-cycloplegic and cycloplegic SE using different agents

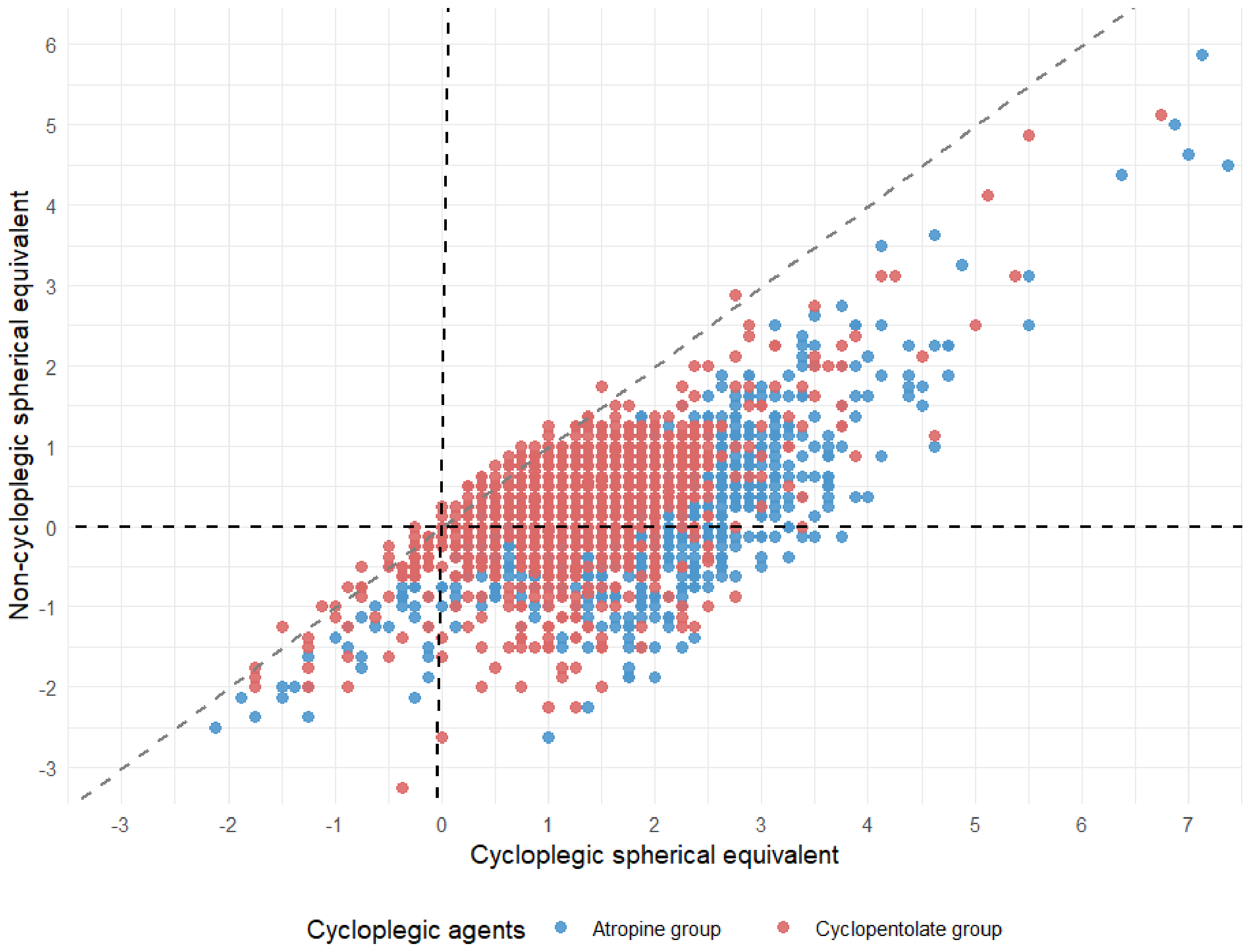

The dashed reference lines in the figure represent Y = 0 (horizontal axis), X = 0 (vertical axis), and Y = X (diagonal) respectively.

© 2025 Wu H et al. *JAMA Ophthalmology*.
