# Supplementary material for: Atropine or Cyclopentolate to Diagnose Premyopia in Preschool Children
Source: JAMA Ophthalmol. 2025 Sep 25;143(11):904–13. doi: 10.1001/jamaophthalmol.2025.3243 (PMC12464850; doi:10.1001/jamaophthalmol.2025.3243)
Supplement: Supplement 2. — Data Sharing Statement. [file jamaophthalmol-e253243-s002.pdf]

Data Sharing Statement

Wu. Atropine or Cyclopentolate to Diagnose Premyopia in Preschool Children. *JAMA Ophthalmol*. Published September 25, 2025. doi:10.1001/jamaophthalmol.2025.3243

Data
Data available: Yes
Data types: Deidentified participant data
How to access data: xianhezi@163.com
When available: With publication

Supporting Documents
Document types: Statistical/analytic code, Informed consent form
How to access documents: xianhezi@163.com
When available: With publication

Additional Information
Who can access the data: researchers whose proposed use of the data has been approved
Types of analyses: myopia-related research
Mechanisms of data availability: after approval of a proposal
